# Educational status as determinant of men’s knowledge about vasectomy in Dangila town administration, Amhara region, Northwest Ethiopia

**DOI:** 10.1186/s12978-017-0314-5

**Published:** 2017-04-18

**Authors:** Abrham Jemberie Temach, Gedefaw Abeje Fekadu, Anemaw Asrat Achamyeleh

**Affiliations:** 1 Aksum University, College of medicine and Health Sciences, Aksum, Ethiopia; 2 Bahir Dar University, College of medicine and Health Sciences, School of Public Health, Bahir Dar, Ethiopia

**Keywords:** Vasectomy, Married men, Knowledge, Dangila town

## Abstract

**Background:**

Although vasectomy is effective and less expensive contraceptive method, only few men are using it in Africa. The main reason for low level use may be low knowledge about vasectomy among men. Only few studies tried to investigate level of knowledge of vasectomy among married men in Ethiopia. But these studies have limitations in measuring knowledge. This study was therefore designed to assess knowledge of vasectomy among married men in Dangila town.

**Method:**

A community based cross sectional study was conducted in Dangila town. Sample size was calculated using OpenEPI online sample size calculator for population based surveys. Multistage sampling technique was employed to recruit the study participants. Data collectors interviewed selected men using structured Amharic questionnaire from June to July, 2014. Two days training was given to data collectors and supervisors. Data were entered and analyzed using SPSS version 16. Binary logistic regression analysis was done to identify determinants of knowledge about vasectomy.

**Result:**

A total of 872 men were interviewed. About 75% of men reported that they had ever heard about vasectomy. Men mentioned friends as main source of information for vasectomy. Among those who had ever heard, only 60.8% defined vasectomy correctly. About 20% defined vasectomy as “it is making the man impotent.” Similarly about 16% equated vasectomy with castration. In this study, only 44.8% of men were knowledgeable about vasectomy. Married men who completed secondary education were 4.10(95%CI; 2.48 – 6.75) times more likely to be knowledgeable about vasectomy compared to those who did not attend formal education. Those who attended above secondary education were 5.73(95%CI 3.76 – 8.73) times more likely to be knowledgeable about vasectomy compared to those who did not attend formal education.

**Conclusion:**

Level of knowledge about vasectomy among married men in Dangla town was low and educational status was an important predictor of knowledge about vasectomy. Efforts are needed to improve knowledge of men about vasectomy. In addition, encouraging boys to complete secondary education may help improve knowledge of men about vasectomy.

## Plain English summary

Vasectomy (male sterilization) is effective, less expensive and easy to perform contraceptive method but only few men are using it in Africa. The main reasons for low level use may be low knowledge about vasectomy. The researchers used community based cross sectional study design with multistage sampling technique to know the level of knowledge of men about vasectomy.

About 75% of men reported that they had ever heard about vasectomy. The respondents mentioned friends as main source of information. Among those who had ever heard, only 60.8% defined vasectomy correctly. The study revealed that only 44.8% of men were knowledgeable about vasectomy. Men who attended modern education were more knowledgeable about vasectomy.

In conclusion, level of knowledge about vasectomy among married men in Dangla town was low. Significant proportion of married men had misconceptions. Educational status was an important predictor of knowledge about vasectomy.

## Background

Ethiopia was the second populous country in Africa with estimated population of 101.7 million in the mid 2016 [1, 2]. The country is also characterized by high maternal and child mortality rates [3–5]. Contraceptive use is effective to balance population growth with development and to reduce maternal and child mortalities [6, 7]. Contraceptive use alone is responsible for about 75% of the global fertility decline [6, 8, 9]. In Ethiopia, contraception had the strongest impact on fertility reduction [10]. Contraceptive use reduced maternal mortality by 44% in 2012. If unmet need was satisfied, the world would have saved additional 29% of maternal deaths [11].

The 1994 International Conference on Population and Development (ICPD) set clear directions to make special efforts to increase men’s responsibility and active involvement in family planning. Yet male involvement in family planning is very low in Africa [12–16]. Ethiopia is one of the countries with substantial increase in contraceptive prevalence rate after the 1990s. But the method mix is dominated by short acting female dependent methods [3, 12].

According to the 2015 estimate, male methods accounted for 21% of contraceptive use worldwide. One of the male methods is vasectomy. Vasectomy is more effective, less expensive and easy to perform [3, 17–19]. But only few African men are using it [3, 19, 20]. Studies in African countries showed that knowledge of vasectomy is low [19, 20]. A qualitative study conducted in Tanzania identified that vasectomy acceptance was limited because of misunderstandings about vasectomy, including fear of decreased sexual performance and other reasons [21]. The 2011 Ethiopian demographic and health survey (EDHS) report indicated that only 11% married women and 17.6% married men heard about vasectomy [22]. A more recent study conducted in Gondar town, Northwestern Ethiopia identified that only 13.3% of married men had good knowledge about vasectomy [23].

The reason for low level of vasectomy use in Ethiopia may be low level of knowledge about vasectomy. There are few studies on knowledge of married men about vasectomy in Ethiopia. The major one is the 2011 EDHS report. But this report assessed only whether men and women had heard about vasectomy. Therefore this study was designed to determine men’s level of knowledge about vasectomy and identify socio demographic factors associated with knowledge about vasectomy among married men in Dangila town, Northwest Ethiopia.

## Methods

The study was conducted in Dangila town administration using community based cross sectional study design. The town is found in Awi administrative zone, Amhara region Northwest Ethiopia. The town administration has 5 urban and 5 rural Kebeles (the smallest administrative unit in Ethiopia) and each kebele has one health post. One health center, two private clinics and 10 health posts provide family planning and other health services in the study area. The aim of the study was to determine the level of knowledge of men about vasectomy and identify socio demographic determinants of knowledge about vasectomy. Sample size was calculated using OpenEpi online sample size calculator tool for population based study assuming that 17% men are knowledgeable about vasectomy, 5% margin of error and design effect of 2. Adding 5% non-response rate, the final sample size was 909. Multistage sampling technique was used to recruit married men for the study. First, five Kebeles were randomly selected from a total of 10. After proportionally allocating the sample size to each selected Kebele (based on the number of married men), households were selected by systematic random sampling technique. The number of households with married men in each kebele was found from the Kebele registration book. Study households were selected from each kebele through systematic random sampling from a random start point. The sampling interval of households in each kebele was determined by dividing the total number of households to the allocated sample size. One married man per household was interviewed. In cases where no eligible men are identified in the selected household, the next eligible household located in the clockwise direction was visited and included.

The interview was conducted using pretested, structured Amharic (the local language) version questionnaire. The questionnaire was prepared in English and translated back to Amharic. Pretest of the questionnaire was done in Fagita Lekoma district (nearby district) and adjustments were made. A value of 1 and 0 was given for each correct and incorrect answer respectively. Frequency and percentage was computed for each knowledge question. For this study, we classified men who correctly answered at least 5 of the knowledge questions knowledgeable and the others less knowledgeable. Data was collected from June to July, 2014 by five grade 12 complete men supervised by two nurses. The data collectors and supervisors were trained for two days on the objective of the study, interviewing techniques, study participant selection and research ethics. Data were entered and analyzed using SPSS version 16. Tables, frequencies and proportions are used to present the data. The association between dependent and independent variables was determined using odds ratio with 95% confidence interval. Logistic regression analysis was performed to control for potential confounders. Ethical clearance was obtained from the Ethical review committee of College of medicine and Health sciences, Bahir Dar University. Written consents were obtained from each participant after explaining the objective of the study.

## Result

### Socio - demographic characteristics of participants

A total of 872 men were included in this study giving a response rate of 95.9%. The mean age of the respondents was 40. 5 years. The majority of men (91.3%) were Orthodox Christians by religion. Most of the respondents were from the Amhara (56.9%) and Awi (41.4%) ethnic groups (Table 1).

**Table 1:** Socio demographic characteristic of married men in Dangila town administration, Northwest Ethiopia, June – July 2014

Socio demographic characteristic	Number (%)
Age (in years)	
• 21–30	167 (19.2)
• 31–40	325 (37.3)
• 41–50	233 (26.7)
• >50	147 (16.8)
Ethnicity	
• Amhara	496 (56.9)
• Awi	361 (41.4)
• Others^a^	15 (1.7)
Religion	
• Orthodox	796 (91.3)
• Muslim	56 (6.4)
• Protestant	20 (2.3)
Educational status	
• Can’t read and write	108 (12.4)
• Read and write	112 (12.8)
• Primary	69 (7.9)
• Secondary	145 (16.6)
• Above secondary	438 (50.2)
Number of live children	
• No child	79 (9.1)
• One to three	546 (62.6)
• Four or more children	247 (28.3)
Occupation	
• Farmer	198 (22.7)
• Merchant	154 (17.7)
• Gov’t employee	424 (48.6)
• Other^b^	96 (11.0)
Residence	
• Rural	198 (22.7)
• Urban	674 (77.3)

Other^a^ refers to Oromo and Tigrie, ^b^daily laborer, unemployed, small business

### Men’s Knowledge about vasectomy

About 75% of men reported that they had ever heard about vasectomy but only 30.7% heard in the past 12 months before the survey. Of those who had ever heard, friends (37.7%), health care providers (21.5%) and radio (12.3%) were the main source of information.

In this study, only 60.8% defined vasectomy correctly. About 20% described vasectomy “it is making the man impotent.” Similarly about 16% defined vasectomy as ‘castration’. In this study, only 44.8% of men were knowledgeable about vasectomy. The proportion of men who responded correctly for each knowledge question was also small (see Table 2).

**Table 2:** Number and percent of married men who gave correct answer for selected questions about vasectomy; Dangila town administration, Northwest Ethiopia, June – July 2014

Knowledge questions	Number (%)
Defined vasectomy correctly	399 (60.8)
Described correctly how vasectomy works	345 (52.6)
Vasectomy is irreversible	442 (67.4)
Vasectomy doesn’t affect sexual desire and performance	229 (34.9)
Time to have vasectomy is when the man wants	511 (77.9)
Vasectomy prevent pregnancy immediately after the procedure is done	153 (23.3)
Vasectomy requires minor surgical procedure	387 (59)
There is no seminal fluid during ejaculation after vasectomy	315 (48)
Vasectomy is done free in Ethiopia	258 (39.3)

### Factors associated with men’s’ knowledge about vasectomy

In the binary logistic regression analysis age, occupation, educational status, residence and number of surviving children showed significant association with level of knowledge about vasectomy among married men in Dangila town. But only educational status remained significant in the multivariable logistic regression analysis (Table 3).

**Table 3:** Determinants of knowledge about vasectomy among married men in Dangila town, Awi zone, Amhara region, Northwest Ethiopia, June – July 2014

Variable	Knowledge about vasectomy		Crude OR	Adjusted OR
	Yes	No		
Age (in years)				
• 21–30	93	74	2.16(1.38 – 3.41) *	1.5(0.81 – 2.76)
• 31–40	141	184	1.32(0.88 – 1.97)	0.90(0.53 – 1.51)
• 41–50	103	130	1.36(0.89 – 2.08)	1.01(0.62 – 1.64)
• >50	54	93	1	1
Ethnicity				
• Amhara	238	258	1	1
• Awi	141	220	0.69(0.53 – 0.92) *	0.75(0.56 – 1.02)
• Others*	12	3	4.34(1.21 – 15.55) *	2.94(0.80 – 10.77)
Religion				
• Orthodox	354	442	1	1
• Muslim	25	31	1.01(0.58 – 1.74)	1.05(0.58 – 1.90)
• Protestant	12	8	1.87(0.76 – 4.63)	1.04(0.41 – 2.69)
Educational status				
• No formal education	39	181	1	1
• Primary	21	48	2.03(1.09 – 3.77) *	1.70(0.90 – 3.23)
• Secondary	74	71	4.84(3.01 –7.78) *	4.10(2.48 – 6.75)*
• Above secondary	257	181	6.59(4.44 – 9.78) *	5.73(3.76 – 8.73)*
Number of live children				
• No child	47	32	1	1
• 1 – 3	266	280	0.65(0.40 – 1.05)	0.87(0.50 – 1.52)
• > = 4	78	169	0.31(0.19- 0.53) *	0.72(0.36 – 1.46)
Occupation				
• Farmer	52	146	1	1
• Merchant	60	94	1.79(1.14 – 2.82)	0.50(0.10 – 2.41)
• Gov’t employee	258	166	4.36(3.00 – 6.33)	0.90(0.18 – 4.53)
• Other**	21	75	0.79(0.44 – 1.40)	0.26(0.05 – 1.33)
Residence				
• Rural	53	145	1	1
• Urban	338	336	2.75(1.94 – 3.90)	1.32(0.29 – 5.99)

Other* refers to Oromo and Tigrie, *means significant at 5%level of significance and 95% level of confidence

Other** refers to daily laborer, unemployed and small business

In this study, married men who completed secondary education were 4.10 (95%CI 2.48 – 6.75) times more likely to be knowledgeable about vasectomy compared to those who did not attend formal education. Similarly, men who attended above secondary level education were about 6 times (95%CI 4.43 – 28.47) more likely to be knowledgeable about vasectomy compared to those who did not attend formal education.

## Discussion

This study attempted to identify the level of knowledge of vasectomy among married men and determinants of knowledge of vasectomy. In this study, about 75% of married men have ever heard about vasectomy. This finding is higher than the 2011 EDHS report which indicated that only 17.6% married men had ever heard about vasectomy [22]. This may be due to time change since EDHS 2011 was done 5 years ago. The other reason is that our study is conducted in more urban setting while EDHS involves both rural and urban areas. Increased effort of governmental and non-governmental organizations to increase acceptance of long acting family planning methods may be the other reason. The data collection method and the curiosity of the data collectors in this study may be the other reason. In the EDHS, the data collectors may be non-health professionals. In addition, information about vasectomy is among the many variables they are collecting. Although the proportion of married men who had heard about vasectomy is high compared to the 2011 EDHS report, the proportion of those who had never heard about vasectomy is not small (24.8%). This indicated that health care workers and the media gave little emphasis for the method. The most frequently mentioned source of information for vasectomy was friends. This reaffirms the above assumption that health professionals and the media gave less emphasis to vasectomy. When friends are source of information, men are less likely to get accurate and full evidence.

Among those who had ever heard about vasectomy, only 60.8% defined it correctly (they reported vasectomy as “contraceptive method by ligating the vas deference”). Other responses in the survey clearly showed the presence of misconceptions about vasectomy among married men in Dangila town. For example, about 65% of married men reported that vasectomy affects sexual desire and performance. A qualitative study in Tanzania revealed that misunderstandings (like vasectomy decreases sexual desire and performance) affected vasectomy acceptance in the country [21]. Biases and misconceptions are the main reasons for low health service use. This is the reason why vasectomy use is low in Ethiopia. Married men thought that vasectomy affect sexual desire and performance because they equate it with castration.

Couples preferring vasectomy as contraception have to avoid unprotected sex or use other methods together with the vasectomy to avoid unwanted pregnancy for the first three months after the procedure. In this study about 23% replied that vasectomy prevents pregnancy immediately after the procedure is done. Vasectomy is simple surgical procedure that can be done even in the physician’s office. But significant proportion (41%) of married men considered it as major procedure that needs several days of admission. All the above findings indicate the presence of knowledge gap about vasectomy among married men in Dangila town.

Currently, family planning services including vasectomy are exempted in Ethiopia. Similarly, vasectomy is provided free for all Ethiopians. But only 39.3% men are aware of this. This fact revealed that married men in Dangila town are less informed about vasectomy. When overall knowledge score was computed, only 44.8% married men were knowledgeable about vasectomy. It can be concluded that this is low as Ethiopia is planning to increase long acting methods use and knowledge is predisposing factor for behavior change.

The study identified educational status as important predictor of better knowledge about vasectomy among married men in Dangila town. This study was consistent with a study done in Gondar town, Northwestern Ethiopia [23]. Married men who completed secondary education were 4.10 (95%CI 2.48 – 6.75) times more likely to be knowledgeable about vasectomy compared to those who did not attend formal education. Similarly, men whose educational status was above secondary were about 6 times (95%CI 4.43 – 28.47) more likely to be knowledgeable about vasectomy compared to those who did not attend formal education. The reason for this is that educated men are more likely to be exposed to different media. They are also more likely to comprehend the information they obtained. Although this study attempted to identify knowledge of men about vasectomy, the study area and variables were small to identify other predictors of knowledge.

## Conclusion

The level of knowledge about vasectomy among married men in Dangla town is low. In this study, educational status was an important predictor of knowledge about vasectomy. Therefore, it is important to increase knowledge of married men about vasectomy through varies information, education and communication methods. Encouraging boys to complete secondary education may help improve knowledge about vasectomy in the future. Further qualitative study triangulated with quantitative design is recommended to identify determinants, biases and misconceptions by increasing the study area and sample size.

## Abbreviations

AOR: Adjusted odds ratio
CI: Confidence interval
EDHS: Ethiopian demographic and health survey
ICPD: International conference on population and development
SPSS: Statistical package for social sciences
